# “Psychiatry is not a science like others” - a focus group study on psychotropic prescribing in primary care

**DOI:** 10.1186/1471-2296-14-115

**Published:** 2013-08-12

**Authors:** Tove M Hedenrud, Staffan A Svensson, Susanna M Wallerstedt

**Affiliations:** 1 Department of Public Health and Community Medicine, Institute of Medicine, University of Gothenburg, P.O. Box 453, Gothenburg, 405 30, Sweden; 2 Angered Medical Centre, Angereds Torg 5, 424 65 Angered, Sweden; 3 Department of Clinical Pharmacology, Sahlgrenska University Hospital, Bruna stråket 21, 413 45 Gothenburg, Sweden

**Keywords:** Prescribing, Psychotropic drugs, Primary health care, Focus groups

## Abstract

**Background:**

Psychotropic drug prescribing is problematic and knowledge of factors affecting the initiation and maintenance of such prescribing is incomplete. Such knowledge could provide a basis for the design of interventions to change prescribing patterns for psychotropics. The aim of this study was to explore the views of general practitioners (GPs), GP interns, and heads of primary care units on factors affecting the prescribing of psychotropic drugs in primary care.

**Methods:**

We performed four focus group discussions in Gothenburg, Sweden, with a total of 21 participants (GPs, GP interns, and heads of primary care units). The focus group discussions were transcribed verbatim and analyzed using manifest content analysis.

**Results:**

Three different themes emerged from the focus group discussions. The first theme S*eeking care for symptoms*, reflects the participants’ understanding of why patients approach primary care and comprised categories such as knowledge, attitudes, and society and the media. The second theme, *Lacking a framework, resources, and treatment alternatives*, which reflects the conditions for the physician-patient interaction, comprised categories such as economy and resources, technology, and organizational aspects. The third theme, *Restricting or maintaining prescriptions*, with the subthemes *Individual factors* and *External influences*, reflects the physicians’ internal decision making and comprised categories such as emotions, knowledge, and pharmaceutical industry.

**Conclusion:**

The results of the present study indicate that a variety of factors may affect the prescribing of psychotropic medications in primary care. Many factors were related to characteristics of the patient, the physician or their interaction, rather than the patients’ medical needs per se. The results may be useful for interventions to improve psychotropic prescribing in primary care.

## Background

The use of psychotropics is increasing both internationally [1] and in Sweden [2], and the majority of these drugs are prescribed in primary care [3-5]. The drug group psychotropics include antidepressants, anxiolytics/hypnotics, mood stabilizers, and antipsychotics, which are all used to treat psychiatric disorders. While the use of psychotropics confers many benefits and is sometimes life-saving, it is also associated with problems of dependency, medicalization and side-effects. Elderly people are particularly sensitive to side-effects, and the risk increases with the number of drugs used. In fact, withdrawal of psychotropics among these patients has been reported to improve cognition and to reduce the incidence of falls [6]. In the Region Västra Götaland in western Sweden, the prescribing of psychotropics is extensive compared to other parts of the country [7]. In this region, a high proportion of elderly people also use three or more psychotropics concurrently, a measure considered an indicator of problematic prescribing [7].

For these reasons, there may be a need for interventions aimed at limiting prescriptions of psychotropics to patients who do not clearly benefit from these drugs. Arguably, as polypharmacy may be a problem in itself, such interventions should target the prescribing of psychotropics in general, rather than that of specific drug groups. Consequently, knowledge is needed about factors that can influence the prescribing of this drug group as a whole.

Previous qualitative studies, however, have only investigated physician decision making concerning specific psychotropic drug groups, such as hypnotics [8], antidepressants [9,10], and anxiolytics [11]. One quantitative study set out to establish if there was an association between physician characteristics (e.g. physician age or practice size) and prescribing of psychotropic medication [12], but the authors concluded that this particular prescribing is hard to predict. Further, previous research has mostly been focused on the initiation of psychotropic drugs. There appears to be a lack of a comprehensive analysis of factors affecting all prescribing of psychotropics in primary care, that is, both initiation and maintenance of prescriptions. Therefore, the aim of this study was to explore the views of general practitioners (GPs), GP interns, and heads of primary care units on factors affecting the prescribing of psychotropic drugs in general in primary care.

## Methods

Focus group interviews are appropriate to use when the goal is to explore people’s knowledge and experiences [13]. Data collection was performed in four focus groups consisting of GPs, GP interns, and heads of primary health care units (Table 1). A GP in Sweden is a physician with a 5-year specialist training in family medicine, whereas a GP intern is a trainee within this specialty. The heads of the units have the overall financial responsibility for a primary care unit, which includes costs for prescribed drugs. Recruitment of participants was performed by two of the authors (S.A.S., S.M.W.), by approaching personal contacts by e-mail and by telephone. In all, 65 persons were approached and 21 agreed to participate and were included in the study (Table 1). The most commonly stated reason for declining participation was a lack of time. A letter was sent 1 week before each focus group discussion was scheduled, containing detailed participant information.

**Table 1:** Characteristics of focus groups

	**Focus group I**	**Focus group II**	**Focus group III**	**Focus group IV**
**Participants**	**GPs**	**Heads of units**	**GPs and GP interns**	**GP interns**
Number of participants (number of women)	4 (1)	5 (5)	5 (3)	7 (1)
Moderator	T.M.H	T.M.H	S.A.S	S.M.W.
Assistant moderator	S.A.S	S.M.W.	T.M.H	T.M.H
Coding of meaning units	T.M.H & S.A.S	T.M.H & S.M.W.	S.A.S & S.M.W.	T.M.H & S.M.W.

*GP* general practitioner.

*T.M.H.* Tove M. Hedenrud.

*S.A.S.* Staffan A. Svensson.

*S.M.W.* Susanna M. Wallerstedt.

The focus group discussions were performed in 2011 at two different health care units in Gothenburg (a city of about 600,000 inhabitants). Apart from the participants and the moderator, one additional researcher took part as an assistant moderator, taking field notes. We chose to alternate between moderators in the focus groups because we believed that our different backgrounds could have an impact on the discussions. The researcher with the most experience of focus groups (T.M.H.: pharmacist, Ph.D.) was moderator twice and the other two researchers acted as moderators once (S.A.S.: GP intern, Ph.D.; S.M.W.: clinical pharmacologist, Ph.D.). The study protocol was approved by the Regional Ethical Review Board in Gothenburg, Sweden.

Each group discussion started with a short, structured introduction by the moderator, and participant information was again distributed to each person together with a consent form to be signed before the discussion started. To facilitate the discussion, we used an interview guide (Table 2) [13], including two fictitious patient cases. Each focus group lasted between 1.5 and 2 hours. The discussions were recorded on a digital voice recorder. After the fourth group, the research group agreed that no new factors had emerged and data saturation was attained.

**Table 2:** Interview guide used in focus groups

*Opening question (to each participant)*	• Please tell us your name and where you work.
*Introductory questions*	• To what extent is the prescribing of psychotropic drugs discussed in your work places?
	• Going back to when you first started to work as a physician (Focus group II: to work in health care), what has been the view on the prescribing of psychotropics through the years?
*Transition questions*	• What are the difficulties with prescribing psychotropics compared to other medicines?
	• The prescribing of psychotropic drugs varies in our country. What is your view on that?
	• What do you think affects the prescribing of psychotropic drugs?
*Key questions*	• Participants were presented with two fictitious case scenarios, concerning patients with complex clinical problems, partly involving psychotropics and were asked to comment.
*Ending questions*	• You have discussed different factors affecting the prescribing of psychotropic drugs. Which factor do you think is the most important?
	• Summary of the discussions by the assistant moderator, followed by the question: How well does this capture what has been discussed?
	• Have we forgotten anything?
	• If time allowed, we asked: We will perform more focus group discussions. In what way do you think the current set-up could be improved?

The focus group discussions were transcribed verbatim and all transcripts were read by all researchers. Names of participants were replaced by codes (fictitious initials). NVivo 9 software was used for data management. We used manifest content analysis, according to Graneheim and Lundman [14], which is an analysis of what the text says rather than what the text talks about (latent content analysis). For each focus group, meaning units were identified, extracted, condensed, and assigned a descriptive code by two researchers independently (Table 1). In the next step, these two researchers met and discussed all codes for that particular focus group until consensus was reached. The three researchers together then sorted the various codes, for all four focus groups, into categories. The categories were discussed at a seminar with other qualitative researchers. As a last step, we identified emergent themes, each one being a thread of an underlying meaning on an interpretative level [14]. Throughout the process of analysis, any disagreements were resolved through discussions.

## Results

**Figure 1:**
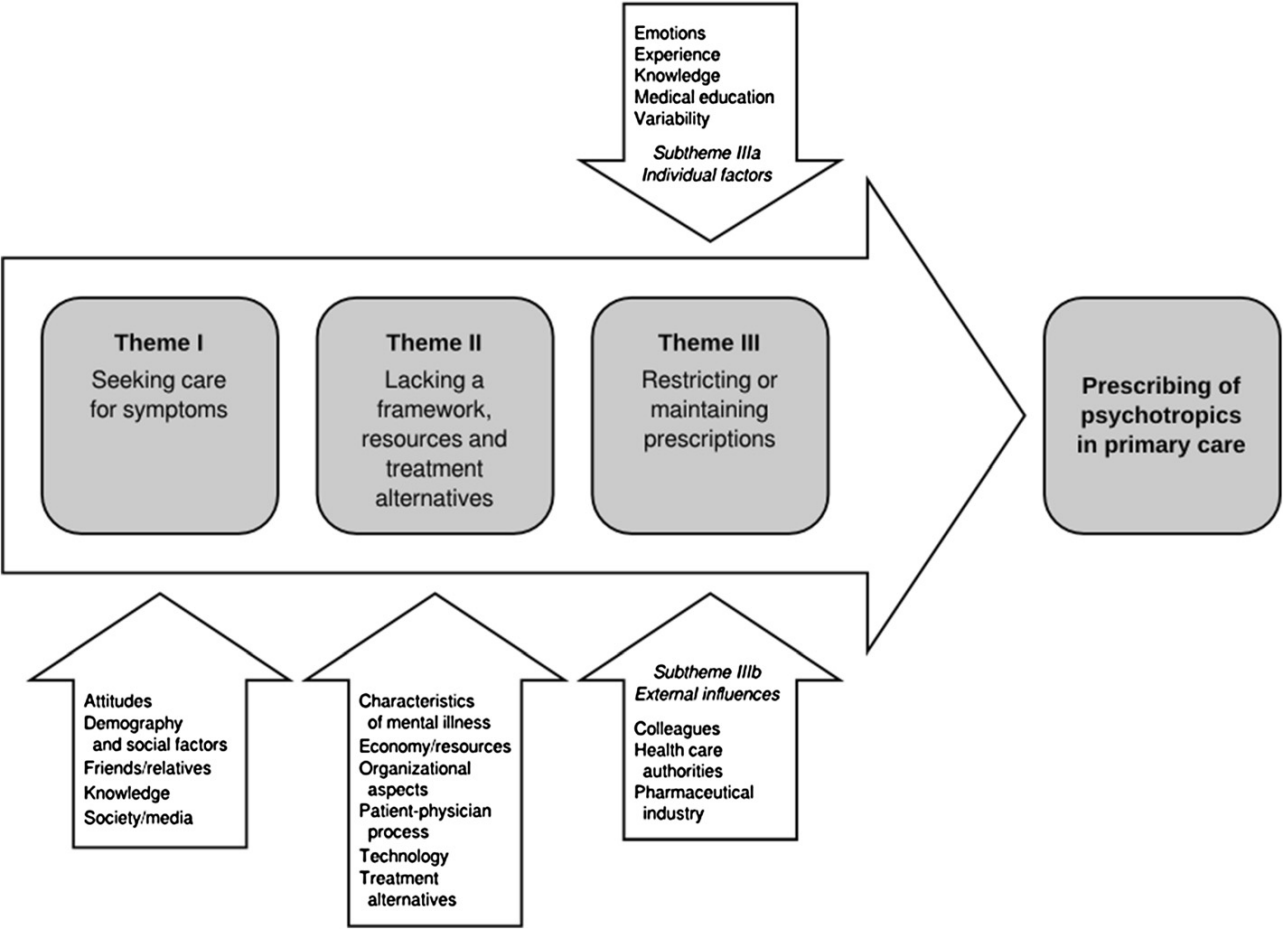
Factors of importance for psychotropic prescribing, resulting themes and categories.

The focus groups and participants are described in Table 1. The heads of the units were all female nurses without the right to prescribe drugs. The rest of the participants were physicians, who, in Sweden, have an unrestricted right to prescribe drugs. In the mixed group (focus group 3), there were 2 GPs and 3 GP interns. Three different themes emerged from the focus groups: S*eeking care for symptoms*; *Lacking a framework, resources, and treatment alternatives*; and *Restricting or maintaining prescriptions.* The latter had two subthemes: *Individual factors* and *External influences*. The themes and categories (in alphabetical order) are presented in Figure 1. The results describe the prescribing of psychotropic medication as a process, from patient care seeking to the actual prescribing. The first theme reflects the participants’ understanding of why patients approach primary care, the second one the conditions for the physician-patient interaction, and the third one the physicians’ internal decision making. All four focus groups contributed to all themes, but for four out of 21 categories there were contributions from only two or three focus groups. The presented quotes were chosen to represent a certain category, that is, to exemplify the discussions in the focus groups.

### Seeking care for symptoms

This theme contains categories describing factors of importance for the decision of the patient to seek care for psychiatric symptoms. According the participants, seeking care was not synonymous, for all patients, with wanting medication.

*Demography and social factors* were considered important for care seeking. Unemployment, divorce, and social problems were brought up as factors of importance. Age was also discussed and the participants meant that youth today cannot handle disappointments and they have high expectations on quick solutions.

“… young people who can’t feel bad for 1 day, just have to get well the same day.” (Focus group II, Head 1)

The participants discussed the importance of *society and the media* in terms of how mental diseases are less stigmatized today. The participants perceived that patients do not consider psychiatric symptoms to be as disgraceful as before, and they are more prone to try a psychotropic medication. They also mentioned that the media are contributing to the idea of antidepressants as “the happy pill” and that there is increased medicalization in society.

“… the main difference that distinguish psychotropics, and psychiatric diseases as well, is that psychiatric diseases have traditionally somehow, for both health care and patients, been kind of shameful. And that is disappearing to some extent.” (Focus group IV, GP intern 4)

Other influences mentioned were positive or negative experiences of medication of *friends and relatives*, but also staff at nursing homes who may demand treatment for a patient who is considered problematic. The impact of *knowledge* (the well-informed patient) and *attitudes* of patients (e.g., a general skepticism against medication) were also discussed.

“So sometimes I meet people who are negative from the start and they are pretty difficult to convince. And sometimes they finally give in and try for a week. And then they go, All right, it didn’t work. Even though I’ve explained that it may take 3 weeks.” (Focus group III, GP 5)

### Lacking a framework, resources, and treatment alternatives

The categories in this theme deal with the conditions of the physician patient consultation. Many of the factors mentioned were considered barriers to rational prescribing.

The category c*haracteristics of mental illness* included difficulties of psychiatric compared with somatic diseases, e.g., diagnosing, where the participants meant there may be a risk of imprecise diagnosing. Further, participants perceived problems in evaluating treatment. The problem of keeping psychiatric treatment evidence-based was also highlighted.

“…psychiatry is not a science like others, not as exact. And it leaves room for many interpretations.” (Focus group I, GP 1)

“It depends a lot on the individual, so it’s hard to comply with general guidelines. Each time you have to manage by trial and error, I would say. And that applies to the drug, the selection, and the dosage as well.” (Focus group IV, GP intern 5)

The *patient physician process* comprised the importance of communication skills, e.g. the ability of the physician to ask the right questions in order to make the right diagnosis. Further, the participants discussed patient factors, such as the patients’ pre-understanding, or fear of adverse effects, as well as their expectations and desires concerning treatment.

“There are lots of people who expect that they have the right to feel well all their lives and that health care should sort it out for them.” (Focus group I, GP 2)

Concerning *economy and resources*, the costs of medication and engaging locum physicians in primary care were mentioned. Further, the lack of time was discussed both with regard to the patient physician consultation and concerning patient follow-up.

Another category was *technology*. According to the participants, technical issues may increase the issue of renewing prescriptions without proper evaluation of the treatment.

“It’s easy to press the button, to click accept.” (Focus group I, GP 1)

(In the multi-dose drug dispensing system in Sweden (ApoDos), all drugs prescribed to an individual can be renewed simultaneously by performing “one click” on the computer. Ordinary prescriptions, on the other hand, need to be renewed one by one. *Authors’ comment*)

Two *organizational aspects* were considered important for the prescribing of psychotropics; namely, level of care and differences between care units. Differences between care units concerned primarily different prescribing traditions at different units. The problem of indeterminate boundaries between psychiatry and primary care, the transfer to primary care of patients previously treated within psychiatric care implicating the inheritance of prescriptions from psychiatry, and the limited possibilities for follow-up in primary care were some of the subcategories of level of care.

“Because then you think, if they end up in the right place perhaps it’ll be the right medication and you’re more in control of things. And that’s what it’s all about. As for me, I feel that a lot of this is an organiza-, well, perhaps that’s stretching it a bit, but to some extent anyway, that this is an organizational issue to a large extent, that there are lots of prescriptions.” (Focus group II, Head 2)

The participants also discussed *treatment alternatives* from two perspectives, namely, effects/side-effects and availability. Based on the perceived effect of treatment, the choice between pharmacotherapy and psychotherapy is influenced by disease severity, as well as patient’s background and age. Further, the participants suggested that limited access to psychotherapy can increase pharmacotherapy treatment.

“No psychoanalyst and no God to offer. We just have that security blanket, the pill, you know…” (Focus group IV, GP intern 6)

### Restricting or maintaining prescriptions

The last theme describes how the prescribing behavior of physicians is influenced by *Individual factors* and *External influences*.

The category *emotions* comprised aspects such as the wish to be, or not to be, updated on new psychotropic drugs, not having the energy to say no to a patient, or the unease about changing drug treatment initiated by colleagues, e.g., specialists in psychiatry:

“It’s much harder to withdraw a drug. And if someone else has started it, I feel, Hang on, I’m stepping on this colleague’s toes.” (Focus group III, GP 6)

*Knowledge*, or, rather, lack of knowledge, was another category described to influence the prescribing of psychotropics. The participants mentioned that primary care physicians do not have enough knowledge about new or specialized psychotropics to prescribe them or to evaluate their effect.

“…we’ve got problems getting help when we feel we’re not quite, don’t have the right competence…//…So I gather we all feel that our knowledge is not sufficient from time to time, for the prescribing we’re doing.” (Focus group I, GP 3)

Participants related the e*xperience* that a physician’s past prescribing behavior may affect his or her future patient clientele. Participants mentioned that low prescribing of benzodiazepines makes those patients seek care elsewhere. Further, the experience of physicians makes them prescribe a small range of psychotropics, i.e., they have their own personal prescribing repertoire.

The impact of *medical education* was also discussed among the participants and many of the participating physicians had gone to medical school at the University of Gothenburg. They said it was their experience that in that academic environment, medication, rather than non-pharmacological treatment, was advocated.

“… those of us who studied in Gothenburg have had it thoroughly hammered in that it’s the drugs that matter.” (Focus group IV, GP intern 7)

*Variability* in prescribing between individuals was mentioned, with several examples of high prescribers given, that is, physicians that the participants had met in practice. Further, psychiatrists as a group were described as prescribing psychotropics to a higher extent and in higher doses compared to GPs. Participants also felt that the prescribing of psychotropics is often arbitrary.

“We just tinker about with the levels of different chemicals. And then we hope and keep our fingers crossed for it to work, and sometimes it’s for the worse.” (Focus group IV, GP intern 8)

External factors of importance are the *pharmaceutical industry*, *colleagues,* and the *health care authorities*.

The category *health care authorities* mainly concerned regional prescribing guidelines and the list of recommended drugs prepared by the drug and therapeutics committees. These publications were mostly regarded as trustworthy by the participants while some colleagues and, in particular, the drug industry were seen as less dependable.

## Discussion

In this focus group study, we explored the views of GPs, GP interns, and heads of primary care units on factors affecting the prescribing of psychotropic drugs in general in primary care. Interestingly, many factors emerged other than the medical needs of the patient; the results indicate that prescribing of psychotropics may be initiated or maintained due to patient or peer pressure, or due to the lack of time or of treatment alternatives. Further, the importance of the characteristics of mental illness was more obvious in our study than in previous research in this area [8-11]. Psychiatry was depicted as a more imprecise field than other specialties. For this reason, psychiatric treatment was considered harder to keep evidence-based, and to require individual considerations to a greater extent. These views on differences between psychiatric and somatic diseases may indicate a perception of lack of scientific knowledge in general in psychiatry, but may also indicate a perceived lack of personal knowledge among the participants.

Emotions were involved in both restricting, and initiating and maintaining prescriptions of psychotropic drugs. The experienced pressure from patients or from colleagues was described in terms of emotions such as not having the energy to argue with the patient, or the fear of stepping on a colleague’s toes. Indeed, previous research has shown that little effort is used to form a second opinion on a patient’s problem when psychotropic drug treatment has been initiated by another doctor [15]. The dilemma of pressure from patients has been described previously [9,15-17], as has the difficulty in finding a balance between rational prescribing and consideration of each patient’s problems [16]. Recently, a hypothesis on emotional prescribing was formulated as an attempt to explain inappropriate or irrational prescribing, and the need for further research on this type of prescribing was emphasized [18].

The problem of indeterminate boundaries between primary care and psychiatry was another recurrent topic. In Sweden, the level of care depends on the type of psychiatric disease as well as the severity of the disease. Thus, psychosis and severe depression are predominantly treated in psychiatry, whereas mild depression and anxiety are often treated in primary care. Clearly, many patients will fall in-between, and it may be difficult to assign these to the appropriate level of care. The participants described referral blocking of patients to psychiatry, on the one hand, and the transfer to primary care of patients previously treated within psychiatric care, on the other. These boundaries create frustration and additional work for the GPs. The takeover of previous prescriptions from specialists has been highlighted previously, but it is not always described as a problem [15]. For instance, it could be perceived as a way to reduce the physician’s responsibility [15]. Also, it may have the positive effect of saving time for the practitioner.

Technology has made it possible to renew a number of prescriptions by using a single mouse click, and doing so may be one way of reducing the perceived high workload of GPs. Indeed, this possibility may increase the overall prescribing of psychotropics, as reported in a recent Swedish study [19]. Further, the time constraints for consultation and their effect on prescribing have been described in previous studies [8,9,11,16]. One study even suggested that changing how doctors use their time would be a better intervention than ’bombarding” them with pharmacological information or encouraging them to change prescribing practices [16].

### Strengths and limitations

We believe that the mix of participants is a strength of the study. The physicians’ workplaces were spread across the city, covering both the city center and the suburbs, thus representing care for different socio-economic groups. Furthermore, the heads of units contributed with a top-down perspective. However, we had no participants from rural areas. In addition, the fact that we recruited by personal contacts may have introduced a source of bias. Thus, there may be some aspects on prescribing of psychotropics that we may not have captured.

In this study, we chose to elicit a discussion about psychotropic prescribing in the widest sense. An alternative strategy would have been to focus on certain drug groups (e.g. anxiolytics), or on specific aspects of prescribing (e.g. initiation or maintenance). Our reasons for this choice was, first, the lack of research on psychotropics as a group, and, second, our belief that a more universal approach will be more useful in the design of interventions aimed at increasing the quality of prescribing.

We chose to use three different moderators and we cannot rule out that this choice may have had an impact on the results. In addition, one of the researchers had the dual role of a GP intern and a researcher. However, the three moderators followed the same interview guide and were all careful not to steer the discussion, e.g., by not responding in an encouraging way to any one of the comments of the participants [13]. We therefore believe that the composition of participants was more important for the dynamics of the discussion in each focus group.

## Conclusions

This study illustrates the complexity of the process of psychotropic prescribing. Numerous factors are important in this process, many of which are unrelated to the patient’s medical needs. The results may be useful when targeting interventions aimed at improved prescribing of psychotropics in primary care.

## Competing interests

The authors report no competing interests.

## Authors’ contributions

TMH, SAS and SMW were all involved in the design, data collection and analysis of data. TMH drafted the manuscript, which was revised by SAS and SMW. All authors have given final approval of the version submitted.

## Pre-publication history

The pre-publication history for this paper can be accessed here:

http://www.biomedcentral.com/1471-2296/14/115/prepub
